# Unfolding Kinetics of a Wormlike Chain under Elongational Flow

**DOI:** 10.3390/polym9060190

**Published:** 2017-05-26

**Authors:** Theo Odijk

**Affiliations:** Lorentz Institute for Theoretical Physics, Leiden University, 2333 CA Leiden, The Netherlands; odijktcf@online.nl; Tel.: +31-71-5145346

**Keywords:** wormlike chain, DNA, unfolding, Stokes flow, fibres, elongational flow

## Abstract

A simple theory of the unfolding kinetics of a semi-flexible polymer chain is presented in terms of a Kramers type picture for the energy of elongation. The hydrodynamic interactions are discussed in terms of slender body theory. It turns out that the elongation of the chain is basically linear in time and independent of the viscosity. The former prediction agrees with experiments on the stretching dynamics of DNA under planar elongational flow. Nevertheless, the theory overestimates the experimental rate by a significant amount for reasons that are unclear.

## 1. Introduction

An unconfined wormlike chain is well known to be characterized by its persistence length $L_{p}$, demarcating the rigid rod limit from that of the fully flexible random coil. It is also generally accepted that $L_{p}$ may no longer be a relevant scale when such a chain is confined. For a worm undergoing elongational flow, one first has to investigate which length scales are relevant in a statistical physical description.

There has been considerable interest in the viscous flow properties of fibers which are often purportedly thought to be elastic rods very weakly perturbed by thermal motion if at all [1]. Buckling of such rods in elongational flows is generally interpreted in terms of classic elasticity theory [2,3,4,5,6]. But another relevant length scale besides the contour length is the persistence length [7,8,9,10,11,12], so slight effects by thermal motion could be discernible even for quite short rods. Furthermore, though global hydrodynamic frictional properties of a rod are simply pinned by the contour length *L*, higher order modes are not. Useful analytical work on the effects of semi-flexibility and hydrodynamics could be carried out in view of the fact that these effects and the inner elastic tension are merely slowly varying variables. This would permit us to understand possible discrepancies between theory and experiment. Another issue is that buckling may be sensitive to the non-uniformity of the elastic properties of the real chain or fiber. Molecular dynamics simulations performed recently on helical polymer chains indicate that the wormlike chain model may have limitations [13]. An investigation of this issue for chains or fibers buckled under elongational flow is warranted, even though they are less heterogeneous than the polymers considered by Palenc̆ár and Bleha [13].

F-actin is a typical biopolymer of choice in experiments involving Stokes flow. Recent work by Strelnikova et al. on this macromolecule in structured microchannels shows that the persistence length is indeed a relevant parameter in elongational flows when the polymer is long enough [14].

There have been many flow experiments on double-stranded DNA because it is readily available in monodisperse form and has been for quite some time. Reese and Zimm studied the fracture of T7 DNA to infer the influence of elongational flow [15]. The distribution of fragmented DNA sections was broad, which they attributed to the presence of folds. This was corroborated by simulations, though the model implemented was rather crude. Somewhat later, Perkins, Smith and Chu presented an exhaustive study of the unraveling [16] of λ-DNA in planar elongational flow. Depending on the initial conditions, the DNA molecules unraveled into dumbbells and half dumbbell structures. Recent work on elongated DNA includes refs. [17,18,19] and an overview [20]. An intriguing study on three types of DNA nanotubes—6-, 8- and 10-helices—focuses on a detailed probabilistic analysis of the fracture caused by elongational forces [21]. The large stiffness of the nanotubes apparently allows for a theory excluding thermal motion.

Qualitative theories for the elongation of polymer chains undergoing thermal motion were already proposed four decades ago [22,23]. A fully quantitative theory exists for Gaussian coils in an equilibrium state [24]. Unfortunately, this is of little use for wormlike chains because we are interested in the elongational dynamics and in the ultimate state the Gaussian limit for the worm breaks down. The uncoiling dynamics has been addressed numerically [25,26] within a free-draining approximation. The tension in a chain is not uniform, an effect that has been investigated both analytically and numerically [27]. These results may be reinterpreted in terms of a slowly varying deflection length adjusting to the nonuniform tension. The extension of DNA in elongational flows was studied by Brownian dynamics simulations, including excluded-volume effects [28], but the two-parameter theory used could break down for wormlike chains, especially at high degrees of elongation.

Here, I present a kinetic theory of the unraveling of a single fold by computing the Kramers energy of the folded chain. This leads to a driving force for the extension. Hydrodynamic interactions are then added on to logarithmic order in a slender body approximation.

## 2. Slender Body Theory for Rods

Let us impose a point-like force F on a bulk fluid at the origin. The fluid is at rest at infinity. The force density is Fδ(R) in terms of a Dirac delta function δ(R), defined in a Cartesian coordinate system (R=x,y,z). The fluid velocity induced by the force is [29,30]

(1) $$\begin{array}{c} v(R)=T\cdot F. \end{array}$$

This is called a stokeslet [30] and the Oseen tensor is given by

(2) $$\begin{array}{c} T=\frac{1}{8\pi \eta }\left(\frac{I}{R}+\frac{\mathrm{RR}}{R^{3}}\right), \end{array}$$

where η is the viscosity of the liquid and I is the unit tensor.

**Figure 1:**
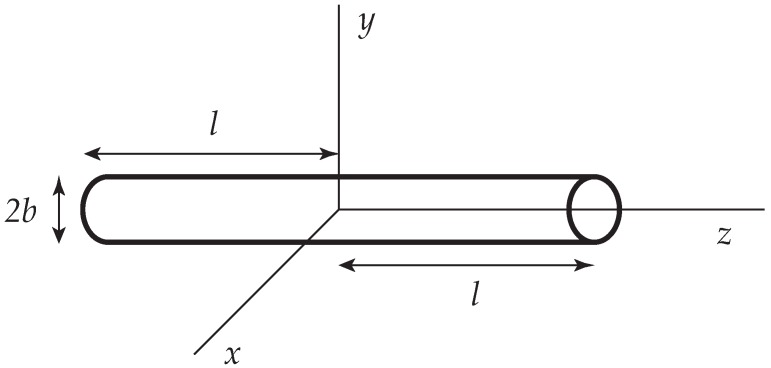
A slender cylinder whose centerline is placed along the *z* axis of a Cartesian coordinate system (x,y,z).

A slender rod of length L=2l and radius *b* (L≫b) is placed at the origin and aligned along the *z* axis (Figure 1). It is then useful to compute the frictional properties of the rod under a variety of flow conditions via the slender body approximation, invoking Equations (1) and (2). A superposition of stokeslets (and possibly additional singularities [30]) is placed along the centerline of the rod and their distribution is chosen in such a way, that the stick boundary on the surface is satisfied as adequately as possible. Within the level of approximations adopted, it is expedient to average the velocity over the circular cross-section and to set this equal to zero [29]. The resulting approximation is often called the Oseen-Burgers procedure. It has been widely used to compute the frictional properties of wormlike and helical wormlike chains [31].

The following elongational flow is now imposed on the rod, under the assumption that the fluid is incompressible.

(3) $$\begin{array}{ccc} v_{x} & = & -\frac{1}{2}\dot{\epsilon }x, \end{array}$$

(4) $$\begin{array}{ccc} v_{y} & = & -\frac{1}{2}\dot{\epsilon }y, \end{array}$$

(5) $$\begin{array}{ccc} v_{z} & = & \dot{\epsilon }z. \end{array}$$

The flow is homogeneous with a constant rate of strain $\dot{\epsilon }$. The center of resistance of the rod may be placed at the origin. Because L≫b, we simply focus on the velocity $v_{z}\left(z\right)$ effective at the centerline given by

(6) $$\begin{array}{ccc} v_{z}\left(z\right) & = & \frac{1}{8\pi \eta }\int_{-l}^{l}dz^{\prime }\;\left[\frac{1}{R}+\frac{{\left(z-z^{\prime }\right)}^{2}}{R^{3}}\right]f\left(z^{\prime }\right),\;\;R^{2}={\left(z-z^{\prime }\right)}^{2}+b^{2}. \end{array}$$

This is an integral equation for the as yet unknown force density f(z) (force per unit length). It can be seen that the kernel leads to an essentially logarithmic modification of *f*. Under the imposed flow expressed by equation (5) at x=0 and y=0, a zero-order estimate for *f* is that it is proportional to *z* [32]. The integrals are evaluated by introducing the variable $\sinh y=(z^{\prime }-z)/b$ and ultimately neglecting terms of O(1). Higher order terms have been derived by Batchelor [32].

(7) $$\begin{array}{ccc} \int_{-l}^{l}dz^{\prime }\;R^{-1} & ≅ & 2\ln\frac{L}{b}, \end{array}$$

(8) $$\begin{array}{ccc} \int_{-l}^{l}dz^{\prime }\;\frac{{\left(z-z^{\prime }\right)}^{2}}{R^{3}} & ≅ & 2\ln\frac{L}{b}. \end{array}$$

To the leading order the force distribution is simply given by

(9) $$\begin{array}{c} f≃\frac{2\pi \eta \dot{\epsilon }z}{\ln L/b}. \end{array}$$

In the hydrodynamic interactions were absent, the force fdz on an infinitesimal segment dz would be proportional to *z* via Equation (5). Equation (9) implies that these interactions simply renormalize the friction by a logarithmic factor. Integration of this equation would give the stress, etc. [32]. Often in the literature, the friction coefficient for translation is substituted for the friction coefficient associated with elongational flows. This is a flawed argument, in principle, although it accidentally gives the correct result to the leading order. In a full analysis, the difference shows up in the correction terms of O(1) [32]. Next, we compute the Kramers energy for a folded chain and account for hydrodynamic interactions as in Equation (9).

## 3. Kramers-Type Theory for Unfolding Dynamics

If we momentarily suppose hydrodynamic interactions may be neglected, we can use a powerful technique introduced by Kramers [33] for setting up theories for elongational flows. The dynamics of a collection of particles as a result of a potential flow is formally equivalent to a problem in equilibrium statistical mechanics. The particles are swept along by a velocity v(r) given in terms of a potential φ(r)

(10) $$\begin{array}{c} v=-\nabla \varphi . \end{array}$$

At the same time, the particles undergo Brownian random forces [33]. Kramers then argued that the system is exactly equivalent to the particles moving under the influence of the potential energy

(11) $$\begin{array}{ccc} U_{k} & = & \sum_{i}\zeta \varphi \left(r_{i}\right), \end{array}$$

with the fluid at rest. Here, the particles are identical and their friction coefficient equals ζ.

Next, we place *N* connected particles ($z_{1}\ldots z_{i}\ldots z_{N}$) along the *z* axis (with $x_{i}$, $y_{i}=0$) in the elongational flow expressed by Equations (3)–(5). With the help of Equations (10) and (11), we have

(12) $$\begin{array}{ccc} U_{k} & = & -\frac{1}{2}\zeta \dot{\epsilon }Z_{c}^{2}-\frac{1}{2}\zeta \dot{\epsilon }\sum_{i}{\left(z_{i}-Z_{c}\right)}^{2}, \end{array}$$

where the center of resistance is given by

(13) $$\begin{array}{c} Z_{c}=\frac{1}{N}\sum_{i}z_{i}. \end{array}$$

Equation (12) implies that the latter is swept along by the fluid and fluctuations relative to $Z_{c}$ determine the configurational statistics of the chain of particles.

**Figure 2:**
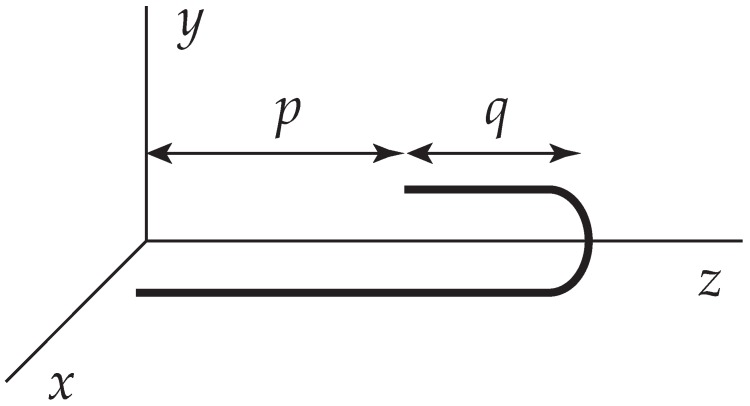
A folded chain whose contour length *L* equals p+2q. The folded section has a length *q*.

Within the Kramers procedure, it is now a simple matter to derive the kinetics of an unfolding chain. We suppose that the elongational flow is so strong, that the semi-flexible chain is effectively almost straight and folded once (Figure 2). Transverse fluctuations may be neglected. The total contour length *L* of the chain is fixed.

(14) $$\begin{array}{c} L=p+2q. \end{array}$$

In the folded configuration, displayed in Figure 2, the center of resistance may be expressed by

(15) $$\begin{array}{c} Z_{c}=\frac{1}{L}\int_{0}^{p+q}ds\;s+\frac{1}{L}\int_{p}^{p+q}ds\;s=\frac{1}{2}L-L^{-1}q^{2}. \end{array}$$

This is the continuum limit of Equation (13). The continuum version of the sum in Equation (12) leads to

(16) $$\begin{array}{ccc} \sum_{i}{\left(z_{i}-Z_{c}\right)}^{2} & = & \frac{1}{L}\int_{0}^{Z_{c}}dt\;t^{2}+\frac{1}{L}\int_{0}^{p-Z_{c}}dt\;t^{2}+\frac{2}{L}\int_{p-Z_{c}}^{p+q-Z_{c}}dt\;t^{2}=\frac{1}{12}L^{2}-q^{2}, \end{array}$$

correct to $O(q^{2})$. In view of Equation (14), *q* must also be small enough, $q<(1-\sqrt{2}/2)L$. Accordingly, the pertinent Kramers energy is

(17) $$\begin{array}{c} U_{k}=\frac{1}{2}k_{1}\eta \dot{\epsilon }q^{2}L. \end{array}$$

We have amended this by a factor involving the hydrodynamic interaction between infinitesimal segments

(18) $$\begin{array}{ccc} k_{1} & = & \frac{2\pi }{\ln\left(\frac{L-q}{b}\right)}+\frac{2\pi }{\ln(q/b)}. \end{array}$$

as in equation (9). There are two force distributions f(z) and g(z) on the two sections of length L−q and *q* respectively, both given by expressions analogous to Equation (6). This neglects the hydrodynamic interaction between the sections but this approximation is discussed below. The constant energy term from Equations (12) and (16) at q=0 must coincide with the energy computed via Equation (9) for a rod of length *L*. This eliminates the unknown friction coefficient ζ. Note that the Kramers energy is virtually proportional to *L*, that is, it is extensive. Apart from the logarithmic factor, $U_{k}$ is basically harmonic. The force exerted on the chain at the fold is now

(19) $$\begin{array}{c} |F_{q}|≅k_{1}\eta \dot{\epsilon }qL \end{array}$$

correct to logarithmic order. On the other hand, the velocity of the shorter section is given by a Stokes approximation for longitudinal translation [29]

(20) $$\begin{array}{c} v_{q}=\frac{|F_{q}|}{k_{2}q\eta } \end{array}$$

with

(21) $$\begin{array}{c} k_{2}=\frac{2\pi }{\ln(q/b)}. \end{array}$$

If we assume that the longer section basically remains stationary, the velocity of unfolding may be written as

(22) $$\begin{array}{c} v_{q}≅\dot{\epsilon }L, \end{array}$$

at small *q*. This result turns out to be independent of the viscosity.

## 4. Discussion

Twenty years ago, Perkins, Smith and Chu [16] presented stretching experiments on λ-DNA with a contour length of L=22 μm in planar elongational flows. The DNA molecules were labeled with a YO-YO dye so that the chain configurations were plainly visible under a microscope. They were able to discern dumbbell configurations, but also singly folded conformations as displayed in Figure 2. The prediction presented here is that the velocity of unfolding should be virtually constant as is indeed borne out by their experiments. Nevertheless, Equation (22) overestimates the rate by an order of magnitude.

It might be thought that the two respective DNA sections within the folded chain are so close together, that this could give rise to an additional frictional force. Let us then estimate the typical distance between the two helical strands. In a recent study [34], two F-actin chains enclosed within a microchannel interacted dynamically as if their mutual interactions were an equilibrium one which is, incidentally, quite surprising. Thus, it is reasonable to balance the deflection interaction [35] against the Kramers energy arising from the flow in the *x* and *y* directions. In the experiments of Perkins et al. [16], the elongational flow is planar, but this will make little difference within an order-of-magnitude argument. If the two DNA chains are imagined to be confined within a tube of diameter *d* that is much smaller than the persistence length $L_{p}$ of the DNA, we have for the total free energy of the two chains

(23) $$\begin{array}{c} F_{\mathrm{tot}}≅k_{3}k_{B}Tld^{-2/3}L_{p}^{-1/3}+k_{2}ld^{2}\eta \dot{\epsilon }. \end{array}$$

Here, $k_{3}$ is a numerical constant, $k_{B}$ is Boltzmann’s constant, and *T* is the temperature. Upon minimizing $F_{\mathrm{tot}}$ with respect to *d*, one obtains

(24) $$\begin{array}{c} \frac{d}{L_{p}}≅W_{i}^{-3/8}, \end{array}$$

where the Weissenberg number pertaining to a persistence segment is defined by

(25) $$\begin{array}{c} W_{i}\equiv \frac{k_{2}\eta \dot{\epsilon }L_{p}^{2}}{k_{3}k_{B}}. \end{array}$$

In Ref. [16], the rate of strain is $\dot{\epsilon }=0.86$ $s^{-1}$, $L_{p}=57$ nm, the viscosity of the sugary buffer is η=41 cP and T=296 K. In a physical sense, the imagined tube is rather “porous”, so $k_{3}$ is not O(1) but rather O(0.1).

The Weissenberg number is $W_{i}=O\left(0.01\right)$ so that $d/L_{p}$ would be O(10), implying $d\gg L_{p}$. Our line of argumentation breaks down. Nonetheless, we may conclude that the two DNA helices within the folded chains are never closer than about $L_{p}$. Any mutual friction is expected to be comparatively weak. In effect, let us suppose some kind of hydrodynamic screening length *h* exists because of the proximity of one section to the other. In an analysis of a suspension of rods at finite concentration undergoing elongational flow, Batchelor computed the screening caused by rods surrounding a test rod [36]. Evidently, the screening that one would have to introduce here for two rods of lengths *q* and L−q is considerably less than that in the case discussed by him that involves many rods close to a test rod. Therefore, *h* is much greater than $L_{p}$. A screening factor incorporated in Equations (1), (2), and (6) would modify the various logarithmic terms only slightly which does not alter Equation (22).

## 5. Concluding Remarks

The Kramers procedure is, of course, well known in the rheological theory of dumbbells [37]. It has been revived here because it leads to a clear picture of the physics involved of a chain unraveling under elongational flow. Hydrodynamic interactions may be introduced later on, a procedure that is correct within the slender body approximation to logarithmic order. The initial stages of unraveling leading to an ensemble of various configurations [15,16,38] have not been discussed at all here. The computations presented only pertain to the later stage of unfolding but they overestimate the experimental rates [16]. On the other hand, theoretical relaxation times estimated for wormlike micelles elongated to folded states are much longer than those measured [39]. These discrepancies need to be addressed.
